# Application Values of 2D and 3D Radiomics Models Based on CT Plain Scan in Differentiating Benign from Malignant Ovarian Tumors

**DOI:** 10.1155/2022/5952296

**Published:** 2022-02-17

**Authors:** Shiyun Li, Jiaqi Liu, Yuanhuan Xiong, Yongzhi Han, Peipei Pang, Puying Luo, Bing Fan

**Affiliations:** ^1^Department of Gynecology, Jiangxi Provincial People's Hospital, The First Affiliated Hospital of Nanchang Medical College, Nanchang 330006, China; ^2^Department of Radiology, Jiangxi Provincial People's Hospital, The First Affiliated Hospital of Nanchang Medical College, Nanchang 330006, China; ^3^GE Healthcare, Hangzhou 310000, China

## Abstract

**Background:**

Accurate identification of ovarian tumors as benign or malignant is highly crucial. Radiomics is a new branch of imaging that has emerged in recent years to replace the traditional naked eye qualitative diagnosis.

**Objective:**

This study is aimed at exploring the difference in the application potential of two- (2D) and three-dimensional (3D) radiomics models based on CT plain scan in differentiating benign from malignant ovarian tumors.

**Method:**

A retrospective analysis was performed on 140 patients with ovarian tumors confirmed by surgery and pathology in our hospital from July 2017 to August 2020. These 140 patients were divided into benign group and malignant group according to the pathological results. The ITK-SNAP software was used to outline the regions-of-interest (ROI) of 2D or 3D tumors on the CT plain scan image of each patient; the texture features were extracted through analysis kit (AK), and the cases were randomly divided into training groups (*n* = 99) and validation group (*n* = 41) in a ratio of 7 : 3. The least absolute shrinkage and selection operator (LASSO) algorithm was used to perform dimensionality reduction, followed by the construction of the radiomics nomogram model using the logistic regression method. The receiver operating characteristic (ROC) curve was drawn, and the calibration curve and decision curve analysis (DCA) were used to evaluate and verify the results of the radiomics nomogram and compare the differences between 2D and 3D diagnostic performance.

**Results:**

There were 396 quantitative radiomics feature parameters extracted from 2D group and the 3D group, respectively. The area under the curve (AUC) of the radiomics nomogram of the 2D training group and the validation group were 0.96 and 0.97, respectively. The accuracy, specificity, and sensitivity of the training set were 92.9%, 88.9%, and 96.3%, respectively, and those of the validation set were 90.2%, 82.6%, and 100.0%, respectively. The AUCs of the radiomics nomogram of the 3D training group and validation group were 0.96% and 0.99%, respectively. The accuracy, sensitivity, and specificity of the training set were 92.9%, 96.3%, and 88.9%, respectively, and those of the validation set were 97.6%, 95.7%, and 100.0%, respectively. DeLong's test indicated that there was no statistical significance between the two sets (*P* > 0.05).

**Conclusions:**

For the differential diagnosis of benign and malignant ovarian tumors, the 2D and 3D radiomics nomogram models exhibited comparable diagnostic performance. Considering that the 2D model was cost-effective and time-efficient, it was more recommended to use 2D features in future research.

## 1. Background

Ovarian cancer is the second most common cause of death from gynecological cancer worldwide [1]. The prognosis of ovarian cancer depends on early diagnosis, surgical treatment, and postoperative systemic treatment. Despite significant progress in the development of treatment methods, the 5-year survival rate after diagnosis of ovarian cancer is only 47%, the 5-year survival rate of patients with stage I ovarian cancer is >70%, and the 5-year survival rate of patients with stage III and IV is <30% [2]. A timely diagnosis of ovarian cancer can improve the survival rate of patients. Ovarian tumor is located in the hidden deep part of the pelvic cavity, without specific clinical symptoms. Ovarian lesions are often detected on CT scans [3].

Radiomics is a new branch of imaging that has emerged in recent years to replace the traditional naked eye qualitative diagnosis. In radiomics, the quantification of imaging data is assisted by various advanced image processing methods [ 4, 5]. Qualitative imaging diagnosis methods are transformed into machine digital quantitative methods [5]. Several studies have shown that CT-based radiomics can efficiently differentiate benign from malignant lesions of kidney, lung, and liver [6]. Thus, it has been hypothesized that the radiomic features of CT images can be used to differentiate benign from malignant lesions of ovarian tumors.

The selection of ROI is critical in the research of radiomics and is directly related to the results of texture feature extraction. The tumor lesions on CT images are displayed in multiple layers, and we can use a single layer (two-dimensional, 2D) of the largest cross-sectional diameter of the tumor or a multilayer (three-dimensional, 3D) of the entire tumor volume to extract the features [6]. Compared with the 3D area-of-interest, the outline of 2D area-of-interest consumes less time and manpower and is simple and fast in the calculation. Compared with the 2D single level, the full level of 3D texture features could provide comprehensive information about the entire tumor [7]. Previous studies have used both 2D and 3D ROI; however, the performance differences between 2D and 3D features show inconsistent results [8, 9]. Additionally, how 2D and 3D features affect the research results as well as their pros and cons are unclear.

The purpose of this study was to explore the performance of radiomics in differentiating benign from malignant ovarian incidental lesions, as well as to compare the performances of 2D and 3D texture features.

## 2. Data and Method

### 2.1. General Information

A retrospective analysis was performed on the clinical and imaging data of 178 patients with suspected ovarian tumors in our hospital from 2017 to 2020. The inclusion criteria were as follows: (a) patients with ovarian tumor confirmed by histopathology; (b) no history of malignant tumors other than ovarian tumor; (c) patients who were undergoing pelvic CT examination within half a month before surgery. Among the patients meeting these criteria, 38 patients were excluded: [1] those who had received radiotherapy, chemotherapy, or radiotherapy-chemotherapy before CT examination (*n* = 20). [2] patients diagnosed with inflammatory diseases (*n* = 11); [3] patients with low image quality (*n* = 7). Finally, our study included 140 patients.

### 2.2. Collection of CT Images

CT images were obtained by dual-source CT (SOMATOM Definition, Siemens) using automatically modulated scanning parameters: tube voltage 120 kV, tube current 150 mA, slice thickness 5 mm, reconstruction interval 1 mm, and slice internal 1 mm.

### 2.3. Segmentation of Area-of-Interest

**Figure 1:**
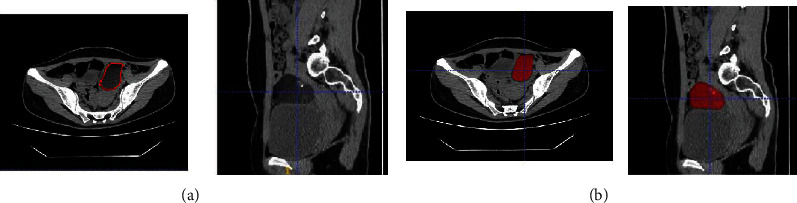
(a) Manual outline of the maximum diameter level of ovarian lesions. (b) Manual outline of the full-level ovarian lesions.

ITK-SNAP (Version 3.6.0) was used to segment all area-of-interests from the baseline DICOM image. The 2D group manually segmented the ROI from the slice with the largest lesion diameter, and the 3D group segmented the full-level ROI of the lesion (Figure 1) [7], which were performed independently by two imaging doctors (A and B, with 5 and 15 years of experience in abdominal imaging, respectively).

### 2.4. Feature Extraction

We used the artificial intelligence life science toolkit (3.0.1.A, GE Healthcare) to extract the texture features of 134 ROIs (62 benign and 72 malignant). For each ROI, 396 features were calculated, including texture, histogram, shape factor, Gray-Level Co-Occurrence Matrix (GLCM), Gray-Level Run-Length Matrix (GLRLM), and Gray-Level Size Zone Matrix (GLSZM). We calculated GLCM and RLM in four directions (0°, 45°, 90°, and 135°) and at three displacements [1, 4, 7] to describe the pattern or spatial distribution of voxel intensity.

### 2.5. Feature Preprocessing

Before feature selection, the features were preprocessed in three steps: [1] the outliers were replaced with the median of the same feature; [2] the control group and the patient group were divided into training group (*n* = 95) and testing group (*n* = 39); [3] *Z*-score normalization was performed on the training dataset to eliminate the difference in the scale of the extracted feature values. The training dataset and test dataset both used the mean and standard deviation calculated using the training dataset alone for normalization. (In the normalization process, the mean was subtracted from the original feature value, and the difference value was divided by the standard deviation).

### 2.6. Feature Selection and Model Establishment

Feature selection and model establishment were performed according to the feature selection and model of the training dataset. First, the robustness and reproducibility of the image features were tested. Since the features were extracted based on the ROI manually segmented by the radiologist, only the most stable features in manual segmentation between different radiologists were used [10]. Using the Spearman rank correlation test, the correlation coefficient of the segmented lesion feature between physician A and physician B was calculated. The features with a correlation coefficient > 0.8 were considered to be robust features. Next, the Max-Relevance and Min-Redundancy (mRMR) algorithm was used, which maximized the relevance between selected features to distinguish between benign and malignant tumors and eliminated redundancy between the features to select features. Second, the least absolute shrinkage and selection operator (LASSO) algorithm was used to adjust *λ* through the penalty parameter to further filter the most useful features. Through ten-fold cross-validation, the optimal *λ* was selected based on the minimum criterion. Then, the radiomic features (Radscore) were calculated for each case, and the selected features were linearly combined and weighted by their respective coefficient factors.

### 2.7. Establishment and Evaluation of Radiomics Nomogram

Univariate logistic regression was used to find independent prediction factors of ovarian tumors. Clinical candidate prediction factors included clinical factors (age, ascites, solidity, and border of tumor cystic), biomarker expression (CA125) [11, 12], and Radscore [13]. Multivariate logistic regression was used to combine these individual prediction factors to develop a more robust malignant prediction model of ovarian tumors and build a radiomics nomogram model [14]. The calibration curve was used for performance evaluation, and the Hosmer-Lemeshow test [15] was used to test the applicability of the model. The receiver operating characteristic (ROC) curve was used to evaluate the diagnostic performance based on the nomogram. The radiomics probabilistic malignancy score of ovarian cancer tumors adopted the nomogram method. According to the ROC curve cutting value, all patients were divided into low probability groups and high probability groups. The ovarian tumor cases of different malignancies were analyzed by nomogram, and its clinical significance was discussed. Additionally, DCA was performed to test the feasibility of the nomogram [16].

### 2.8. Statistical Analysis

Kolmogorov-Smirnov test was used for data normalization. SPSS 19.0 software, and R statistical tools (v3.4.4) were used. The *t*-test (normally distributed data) and Mann–Whitney *U* test (skewed distribution data) were used to count the probability scores of radiomics of benign and malignant samples, where the scores were expressed as mean ± standard deviation (*x* ± *s*). The *χ*^2^ test was used to compare the two sets of count data. The predictive effect of each model was evaluated using typical diagnostic indicators, such as accuracy, sensitivity, specificity, and area under the curve (AUC) [17].

## 3. Results

### 3.1. Case Characteristics

The factors such as age, ascites, boundary, cystic-solid tumor, or biomarker expression (i.e., CA125) were included in the multivariate logistic regression analysis (Table 1). In the training set, age, ascites, cystic-solid tumor, and CA125 were found to be independent clinical prediction factors (Table 2).

### 3.2. Establishment and Evaluation of Clinical Prediction Model

**Figure 2:**
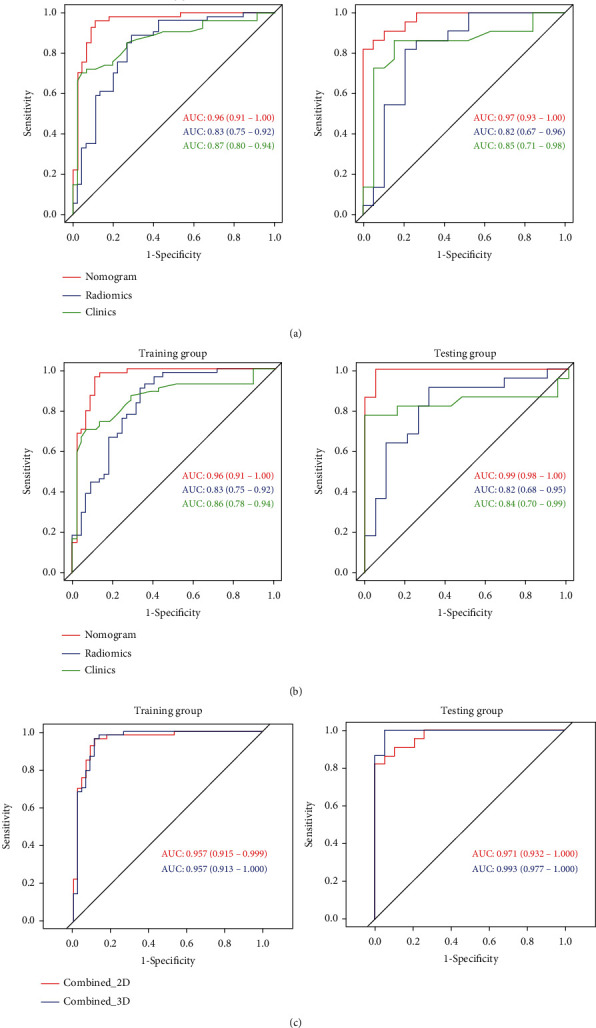
(a) ROC of 2D clinical, radiomics, and nomogram models to differentiate benign from malignant ovarian tumors. (b) ROC of 3D clinical, radiomics, and nomogram models to differentiate benign from malignant ovarian tumors. (c) AUC of 2D and 3D nomogram models to differentiate benign from malignant ovarian tumors (*P* < 0.05).

The constructed clinical prediction model for differentiating benign from malignant ovarian tumor found the following performance indicators. In the 2D training set, the AUC was 0.87 (95% CI: 0.80-0.94), and the sensitivity, specificity, and accuracy were 70.4%, 75.6%, and 81.8%, respectively. The AUC of the validation set was 0.85 (95% CI: 0.71-0.98), and the sensitivity, specificity, and accuracy were 72.7%, 94.7%, and 82.1%, respectively. The AUC of the 3D training group and the validation group were 0.86 (95% CI: 0.78-0.94) and 0.84 (95% CI: 0.70-0.99), and the sensitivity, specificity, and accuracy in the two groups were 70.4% and 77.2%, 90.3% and 84.2%, and 80.8% and 80.5% (Figure 2, Table 3).

### 3.3. Establishment and Evaluation of Radiomics Prediction Model

**Figure 3:**
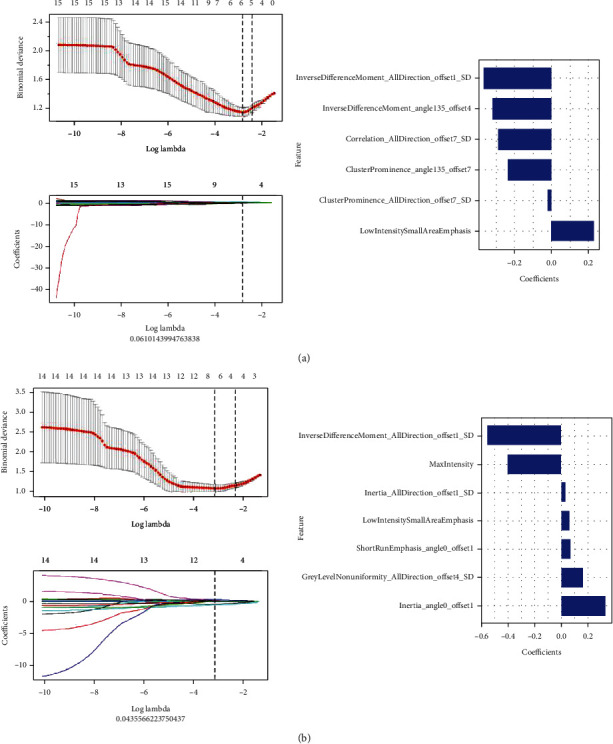
The feature selection process, selected radiomic features, and corresponding coefficients of radiomics model: (a) 2D model and (b) 3D model.

The 2D selected 6 features, 3D selected 5 features, and these features were used to construct 2D and 3D radiomics models (Figure 3). The AUC of 2D training group and 2D validation group was 0.83 (95% CI: 0.75-0.92) and 0.82 (95% CI: 0.67-0.96); the accuracy, specificity, and sensitivity of 2D training group and 2D validation group were 80.8% and 80.5%, 71.1% and 78.9%, and 88.9% and 81.8%; the AUC of 3D training group was 0.83 (95% CI: 0.75-0.92); the sensitivity, specificity, and accuracy were 90.7%, 66.7%, and 79.8%, respectively; the AUC of 3D validation group was 0.86 (95% CI: 0.68-0.95); the sensitivity, specificity, and accuracy were 81.8%, 73.7%, and 78.0%, respectively (Figure 2, Table 3).

### 3.4. Establishment and Evaluation of Radiomics Nomogram Models

**Figure 4:**
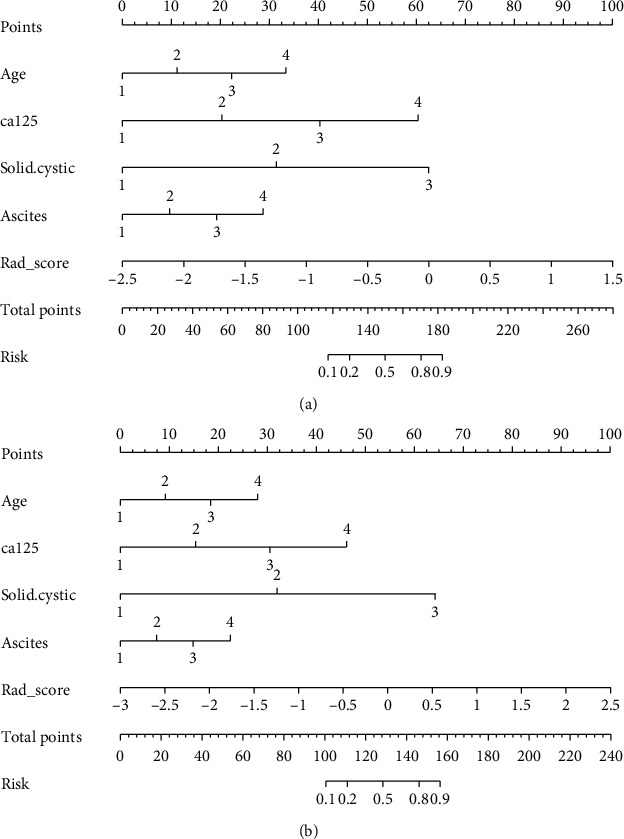
Radiomics nomogram ((a) 2D model and (b) 3D model).

The results of univariate logistic regression analysis showed that age, ascites, cystic-solid, CA125, and radiological signs could independently predict and diagnose ovarian tumors (Table 2). These prediction factors could construct a more stable radiomics nomogram prediction model through multivariate logistic regression analysis (Figure 4).

The patient calibration curve showed that the predicted ovarian tumor types were consistent with the actual types of ovarian tumors. The 2D radiomics nomogram predicted the AUC of benign and malignant ovarian occupancy in the training group and the validation group to be 0.96% (95% CI: 0.91-1.00) and 0.97% (95% CI: 0.93-1.00), respectively. The accuracy, specificity, and sensitivity of the training set were 92.9%, 88.9%, and 96.3%, respectively, and those of the validation set were 90.2%, 82.6%, and 100.0%, respectively. The 3D radiomics nomogram predicted the AUC of benign and malignant ovarian occupancy in the training group and the validation group to be 0.96% (95% CI: 0.91-1.00) and 0.99% (95% CI: 0.98-1.00), respectively. The accuracy, sensitivity, and specificity of the training set were 92.9%, 96.3%, and 88.9%, respectively, while those of the validation set were 97.6%, 95.7%, and 100.0% (Figure 2, Table 3).

**Figure 5:**
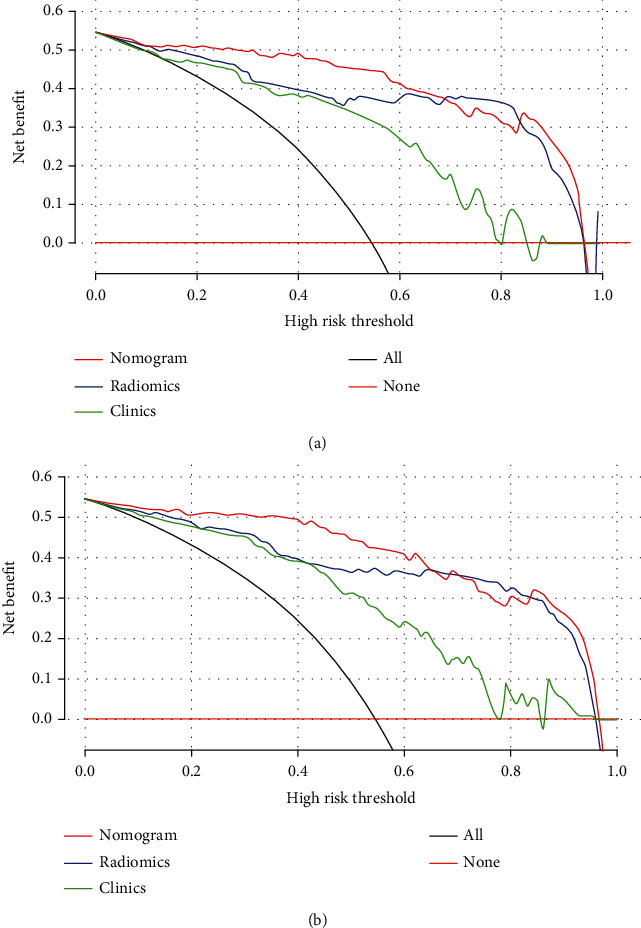
DCA of (a) 2D and (b) 3D radiomics models and clinicopathological features (green, blue, and dark red lines correspond to clinical, radiomics, and nomogram model, respectively). Light gray lines show that all radiomics models, and clinicopathological features were related to hypotheses related to malignant ovarian tumors. Additionally, the dark red lines show that all radiomics models and clinicopathological features were related to hypotheses not related to malignant ovarian tumors.

DeLong's test revealed that in the 2D and 3D training and test sets, the AUC of the clinical information-based model was significantly different from that of the nomogram-based model. The differences between the clinical information model and the pure radiomics model were not statistically different, and there is no statistically significant difference in the differential diagnostic performances between the 2D and 3D nomogram model. Hence, 2D and 3D nomogram models exhibited good performances (Table 4). Additionally, the Hosmer-Lemeshow test demonstrated that there was no statistically significant difference between the training and test subsets (*P* > 0.05). This verified the superiority of the nomogram diagnosis. The nomogram was also used to estimate the probability score of ovarian tumors. According to Youden index 19 (2D cut-off value was 0.415, and 3D cut-off value was -0.107), patients were divided into low probability group and high probability group, and the index was defined according to the nomogram of the training set. The high probability group and the low probability group had significant differences in the number of benign and malignant samples (*P* < 0.0001).  Figure 5 depicts the DCA of 2D and 3D radiomics nomograms. The radiomics and nomogram methods were superior to the clinical models of the “no treatment” and “treatment all” strategies. The 2D and 3D models had treatment probability thresholds ranging from 0 to 0.7 (Figure 5).

## 4. Comment

### 4.1. Principal Findings of the Study

In this study, the 3D radiomics model and radiomics nomogram model of patients with ovarian tumors were constructed and compared with the 2D model. In the training group and the validation group of the 3D model, the AUC of the radiomics nomogram model was 0.96% (95% CI: 0.91-1.00) and 0.99% (95% CI: 0.98-1.00); the radiomics nomogram model had extremely high sensitivity and specificity. Compared with the other two models, the difference was statistically significant. The radiomics model had high sensitivity, and the clinical model had high sensitivity. The results were consistent with the evaluation results of the 2D model. In the horizontal comparison, the 2D and 3D models had the same diagnostic efficiency for the evaluation of benign and malignant ovarian incidental tumors, and the difference was not statistically significant.

ROI is sometimes performed on a single section with the largest cross-sectional diameter (2D) of the tumor and sometimes on multiple sections or the entire tumor volume (3D). A 3D model based on the full volume includes the entire tumor lesion and can extract more meaningful features for research and analysis compared with the 2D model that includes only studies the largest cross-sectional area. Machado et al. [9] found that radiomics could predict the recurrence of nonfunctional pituitary adenomas after the first operation. Compared with 2D radiomic features, 3D radiomics has a better discriminative ability. Ng et al. [18] explored whether there was a difference between the maximum cross-sectional area enhanced CT texture feature and the whole tumor enhanced CT texture feature and its predictive effect on the clinical prognosis. The results showed that the whole tumor analysis could effectively reflect tumor heterogeneity.

### 4.2. Clinical Implications

Since radiomic features were extracted from the ROI, manual outline required the researcher to have relevant professional knowledge and spend a considerable amount of time, and the calculation of features needed extensive calculations [17]. This was particularly prominent in the research based on 3D images, which restricted the development of related fields. However, the research showed that it was not clear whether the extra time and labor related to capacity evaluation was necessary [19]. Hence, as another trade-off, the time-saving 2D model was also used in some research, which was performed at a single axial level, usually the largest cross-section of the lesion, rather than the entire tumor. Whether this is representative of the entire tumor is still unknown. In some research, the 2D model has shown better performance, and this seems quite counter-intuitive. Meng et al. [20] found that the model built with 2D radiomic features showed comparable performance to the model built with 3D features in terms of characterization of the prediction of lymphatic and vascular metastasis of gastric cancer and believed that the time-saving 2D model would be a better choice for studying gastric cancer. Shen et al. [21] compared the difference between 2D and 3D image features of CT images and prognostic performance of nonsmall cell lung cancer and found that image features had a certain predictive effect on the prognosis of nonsmall cell lung cancer; however, the 2D features showed better performance. Thus, based on the calculation cost of radiomic features, the use of 2D features was recommended.

In the feature selection, among the final features extracted by the 3D model, there were two features that were the same as those extracted by the 2D model, namely, the inverse different moment and the advantage of the low-intensity small area. The feature with the largest negative coefficient value was also the inverse different moment, indicating that the element values of benign ovarian tumors were more uniform than those of malignant tumors. The radiomic features were very stable and well represented the texture of the tumor, which had a good reference value for the differentiation of benign from malignant ovarian tumors. The feature with the largest positive coefficient value was the GLCM inertia, which belonged to the GLCM feature. It reflected the clarity of the image and the depth of the texture groove. Contrast was directly proportional to texture grooves; the larger the value of the groove, the clearer the image. On the contrary, the smaller the value of the groove, the smaller the image contrast and the more blurred the image [22]. GLN measured the similarity of the gray-level intensity values in the ROI. The larger the feature value, the higher was the repetition frequency of a gray-level value in the matrix, which indicated that the distribution of gray value was more uniform [23]. SRE measured the distribution of short run-length in the image matrix. The larger the feature value, the shorter was the run-length, with a finer image texture [24]. These two reflected the judgment value of heterogeneity of ovarian tumors in the differential diagnosis of benign and malignant tumors. Unlike the 2D model, the first-order statistical features (maximum intensity and statistical parameters) were screened out in the 3D model that was related to the attributes of a single pixel, and the distribution of voxel intensity in CT images was described by common basic metrics [25]. However, 2D and 3D models had not screened out the morphological features, which mainly described the 3D size of ROI, spatial geometric shape characteristics and did not reflect the heterogeneity within the image. The results of the study showed that the nature of the ovarian tumor was not closely related to the size and shape of tumors. Vos et al. [26] proposed that all tumor lesions were small tumors at the beginning, showing short and stable growth, and the size of the tumor depended on the measurement time. Hence, small or medium tumors were not reliable biomarkers.

### 4.3. Strengths and Limitations

In this study, 2D and 3D image features had comparable predictive performance on the nature of the ovarian tumor. Analysis suggested that the 3D model brought more noise in the delineation of ROI and feature selection, drowning out effective information and disturbing research results [20]. There were two main sources of noise. First, the delineation of the ROI of the target lesion was a subjective and subtle process. Different doctors might have different opinion in judging whether there was a disease and determining the location of the boundary of the disease. Even if it passed the consistency analysis, it still could not avoid the limitations of naked eye observation and the interference of personal subjectivity. Compared with the 2D mode, the 3D mode was more susceptible to influence because its multilevel outline magnified the influence of factors. Second, the noise was related to the thickness of the scan layer. For different images, different CT scanners had different reconstruction schemes, and their thickness was also different. Since multilayer or 3D ROI had multiple blurred lesion boundaries at different layers and high sensitivity to different thicknesses, its signal-to-noise ratio could have been lower than that of single-layer 2D ROI.

### 4.4. Conclusions

The proposed 2D and 3D radiomics models had the same diagnostic efficiency for the differentiation of benign and malignant ovarian tumors. However, considering better clinical applicability and lower cost of manpower and material resources, the 2D model was recommended for follow-up research.

## Figures and Tables

**Table 1:** Case distribution in the training set and validation set.

	Training group (*n* = 99)			Validation group (*n* = 41)			*P* value
	Benign	Malignant	*P* value	Benign	Malignant	*P* value	
Quantity	45	54		19	22		
Age (year)			0.0164			0.0015	0.3032
<18	4 (8.9%)	1 (1.9%)		1 (5.3%)	0 (0.0%)		
>18, ≤30	7 (15.6%)	2 (3.7%)		8 (42.1%)	0 (0.0%)		
>30, ≤50	20 (44.4%)	20 (37.0%)		6 (31.6%)	7 (31.8%)		
>50	14 (31.1%)	31 (57.4%)		4 (21.1%)	15 (68.2%)		
CA125 (U/mL)			<0.0001			0.0009	0.8859
<35	22 (48.9%)	4 (7.4%)		9 (47.4%)	4 (18.2%)		
>35, ≤200	20 (44.4%)	16 (29.6%)		10 (52.6%)	5 (22.7%)		
>200, ≤500	2 (4.4%)	11 (20.4%)		0 (0.0%)	4 (18.2%)		
>500	1 (2.2%)	23 (42.6%)		0 (0.0%)	9 (40.9%)		
Ascites			<0.0001			0.0004	0.4627
None	26 (57.8%)	10 (18.5%)		15 (78.9%)	4 (18.2%)		
Small	17 (37.8%)	12 (22.2%)		3 (15.8%)	4 (18.2%)		
Moderate	0 (0.0%)	13 (24.1%)		1 (5.3%)	4 (18.2%)		
Large	2 (4.4%)	19 (35.2%)		0 (0.0%)	10 (45.5%)		
Cystic-solid tumor			<0.0001			0.0044	0.6431
Cystic	17 (37.8%)	2 (3.7%)		7 (36.8%)	0 (0.0%)		
Cystic-solid	26 (57.8%)	45 (83.3%)		12 (63.2%)	20 (90.9%)		
Solid	2 (4.4%)	7 (13%)		0 (0.0%)	2 (9.1%)		
Boundary			<0.0001			0.4082	0.3399
Clear	41 (91.1%)	24 (44.4%)		16 (84.2%)	15 (68.2%)		
Blur	4 (8.9%)	30 (55.6%)		3 (15.8%)	7 (31.8%)		
Radscore median [iqr]	-0.6 [-1.1, 0.6]	0.8 [0.6, 1.1]	<0.0001	-0.6 [-1.0, -0.1]	0.8 [0.4, 1.0]	0.0005	0.7503

**Table 2:** Univariate and multifactor logistic regression analysis of benign and malignant prediction factor of ovarian tumor.

	Univariate regression analysis			Multifactor regression analysis		
Variables	OR	(95% CI)	*P* value	OR	(95% CI)	*P* value
Age	2.340	[1.331; 4.112]	<0.001	2.48	[1.07; 5.74]	0.034
CA125	5.225	[2.701; 10.110]	<0.001	3.53	[1.61; 7.73]	0.002
Boundary	12.813	[4.023; 40.810]	<0.001			
Cystic-solid	7.262	[2.423; 21.762]	<0.001	6.37	[1.75; 23.22]	0.005
Ascites	3.558	[2.091; 6.054]	<0.001	2.29	[1.21; 4.34]	0.011

**Table 3:** Predictive performances of a clinical model, radiomics model, and radiomics nomogram model.

Group	Model	Accuracy	95% CI	Sensitivity	Specificity
Training group (2D)	Clinical	0.818	[0.728; 0.889]	0.704	0.956
	Radiomics	0.808	[0.717; 0.880]	0.889	0.711
	Nomogram	0.929	[0.860; 0.971]	0.963	0.889
Validation group (2D)	Clinical	0.829	[0.679; 0.928]	0.727	0.947
	Radiomics	0.805	[0.651; 0.912]	0.818	0.789
	Nomogram	0.902	[0.769; 0.973]	1.000	0.826
Training group (3D)	Clinical	0.808	[0.717; 0.880]	0.704	0.933
	Radiomics	0.798	[0.705; 0.872]	0.907	0.667
	Nomogram	0.929	[0.860; 0.971]	0.963	0.889
Validation group (3D)	Clinical	0.805	[0.651; 0.912]	0.772	0.842
	Radiomics	0.780	[0.624; 0.894]	0.818	0.737
	Nomogram	0.976	[0.871; 0.999]	0.957	1.000

**Table 4:** DeLong's test of a clinical model, radiomics model, and nomogram model.

Group	Model 1	Model 2	*P* value
Training group (2D)	Clinical	Radiomics	0.538
	Radiomics	Nomogram	0.001
	Nomogram	Clinical	0.007
Validation group (2D)	Clinical	Radiomics	0.779
	Radiomics	Nomogram	0.020
	Nomogram	Clinical	0.042
Training group (3D)	Clinical	Radiomics	0.629
	Radiomics	Nomogram	0.002
	Nomogram	Clinical	0.006
Validation group (3D)	Clinical	Radiomics	0.811
	Radiomics	Nomogram	0.010
	Nomogram	Clinical	0.039

## Data Availability

The data that support the findings of this study are available from the corresponding author upon reasonable request.

## Abbreviations

CT: Computed tomography
AUC: Area under the curve
ROC: Receiver operating characteristic
AK: Analysis kit
LASSO: The least absolute shrinkage and selection operator
DCA: Decision curve analysis.
